# Unresectable hepatic PEComa: a rare malignancy treated with stereotactic body radiation therapy (SBRT) followed by complete resection

**DOI:** 10.1186/s13014-018-0974-5

**Published:** 2018-02-20

**Authors:** Simon Kirste, Gian Kayser, Anne Zipfel, Anca-Ligia Grosu, Thomas Brunner

**Affiliations:** 1 Department of Radiation Oncology, University Medical Center Freiburg, Robert-Koch-Str. 6, 79106 Freiburg, Germany; 2 Department of Surgical Pathology, University Medical Center Freiburg, Faculty of Medicine, Freiburg, Germany; 3 German Cancer Consortium, Partner Site Freiburg (DKTK), Freiburg, Germany

**Keywords:** Malignant perivascular epithelioid cell tumor, PEComa, Liver, SBRT, Neoadjuvant, Resection

## Abstract

**Background:**

Perivascular epithelioid cell tumors (PEComas) are rare mesenchymal tumors occurring in various anatomic regions. Although diagnostic criteria and treatment management are not established, current treatment options consist of surgery and chemotherapy including mTOR inhibitors.

Stereotactic body radiation therapy (SBRT) is a non-invasive ablative treatment which has shown excellent control rates for more common types of unresectable liver tumors and metastases. In this report we present a rare case of PEComa of the liver that was treated by stereotactic radiotherapy followed by resection. Staging and evaluation of treatment response was done by FDG-PET/CT. This case highlights the potential of SBRT as a neoadjuvant treatment even for rare liver malignancies. It is the first case of liver PEComa treated by SBRT and resection.

**Case presentation:**

A 52-year-old woman presented at an external hospital with abdominal pressure and pain in the right upper abdominal quadrant. A CT scan showed a 700 cm^3^ liver lesion in segment IV. In repeated biopsy in July 2015 histopathological workup showed a pleomorphic epitheloid tumor with small to medium sized cells expressing vimentin and melan-A while being negative for cytokeratin establishing the diagnosis of PEComa of the liver.

To achieve high, ablative doses a stereotactic body radiotherapy (SBRT) technique was chosen consisting of 60Gy (biologically effective dose 105Gy) in 8 fractions of 7.5Gy. Radiotherapy planning was based on MRI resulting in a planning target volume (PTV) of 1944 cm^3^. Treatment toxicity was limited to a slight elevation of transaminases (grade 1 and 3). A complete resection was performed 21 weeks after radiotherapy confirmed by negative surgical margins.

At last follow-up 21 months after therapy, MRI showed neither local nor distant tumor recurrence. The patient was in stable condition (ECOG 1) and without late radiation toxicity.

**Conclusions:**

This is the first documented case of liver PEComa treated by SBRT and resection. A favorable post-treatment course demonstrates that SBRT is a potential neoadjuvant treatment that is capable of reducing an inoperable rare liver tumor to a resectable lesion.

## Background

Perivascular epithelioid cell tumors (PEComas) are rare mesenchymal tumors for which diagnostic criteria and treatment management are not yet established [1]. The term PEComa was first introduced in 1996 by Zamboni and Bonetti who suggested the name PEComa for neoplasms composed of a pure proliferation of perivascular epithelioid cells (PEC) [2]. Histological hallmarks are the presence of a distinct cell type, known as “perivascular epitheloid cell”, and the coexpression of smooth-muscle (actin and/or desmin) and melanocytic markers (HMB-45 and/or melan A) [3]. The etiology of PEComas remains uncertain. They predominantly affect women and occur in nearly every organ [4]. The incidence of benign and malignant PEComa of the liver is much lower than for other locations [5].

In the WHO classification of soft tissue and bone sarcomas (2002), PEComas are categorized in the subgroup of “malignant tumors with uncertain differentiation” [1]. To date, PEComa classification encompasses angiomyolipomas (AML), lymphangioma, lymphangioleiomyomatosis (LAM), clear cell sugar tumors (CCST) and not otherwise specified PEComas (NOS) [3]. PEComas manifest different biological behavior with approximately one-third presenting with locally aggressive behavior (malignant PEComa) [6].

Another characteristic of PEComas is their relation to the tuberous sclerosis complex (TSC). Similar gene mutations in TSC1 and TSC2 genes have been demonstrated in a number of PEComas. TSC genes seem to have an important role in the regulation of the mTOR pathway. Selectively inhibiting mTOR pathways has shown efficacy in malignant PEComa [7].

**Fig. 1:**
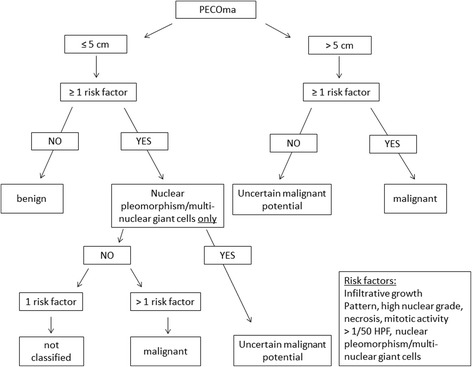
Malignancy classification adapted from Folpe et al. [2]

Folpe et al. developed a risk stratification based on pathological findings and risk factors to classify PEComa into “benign”, “of uncertain malignant potential” and “malignant” (Fig. 1) [2].

Neither diagnostic criteria nor treatment management are established. The definitive diagnosis is obtained by biopsy. Imaging is used to determine the local extent and for staging purposes [8]. Although pathognomonic imaging-based features of PEComas have not been defined, most commonly used imaging methods include computer tomography (CT) and ultrasound. PET/CT has no role in the diagnosis of PEComa. The mainstay of therapy is resection. Some PEComas are adequately treated by complete resection; however, the therapy management of tumors of unknown malignant potential or inoperable tumors remains unclear. Adjuvant treatment in addition to surgery has been reported for only a few patients. An extensive review of treatment strategies of 234 cases reported that nearly all patients were treated with surgery; only 2 patients presented with unresectable disease [4]. Very few (6 cases) received neoadjuvant treatment (radiotherapy alone 2 patients, chemotherapy alone 3 patients and radiochemotherapy 1 patient) and only 18% of patients received adjuvant therapy after resection (8 patients radiotherapy, 2 patients radiochemotherapy). The review highlighted the diversity of treatment protocols, histologic heterogeneity and diverse follow up intervals, as well as the natural history of the disease. Based on this review no final conclusions or treatment recommendations can be given. Inoperability of the tumor was generally associated with a poor outcome. Four out of five cases of unresectable PEComa due to tumor size or metastatic disease proceeded fatal, one case of malignant epitheloid angiomyolipoma showed a good response to systemic therapy with an mTOR-inhibitor for 7 months of follow-up [9–13].

The role of mTOR-inhibitors in the treatment of malignant PEComa is not clear. mTOR-inhibitors are recommended for treatment of TSC associated renal angiomyolipoma based on three clinical trials reporting a prolonged response rate in approximately 58% of patients with renal and associated hepatic AML [14–16].

We report on a case of inoperable hepatic PEComa that was treated by neoadjuvant radiotherapy followed by resection. FDG-PET/CT was used for staging and evaluation of treatment response.

## Case presentation

A 52-year-old woman presented at an external hospital with a chief complaint of abdominal pressure and pain in the right upper abdominal quadrant. A CT scan revealed a 700 cm^3^ liver lesion in segment IV. Hepatocellular carcinoma, metastasis or cholangiocellular carcinoma were excluded by biopsy but no definite diagnosis could be established. Subsequently, she was referred to our cancer center for further evaluation and management.

**Fig. 2:**
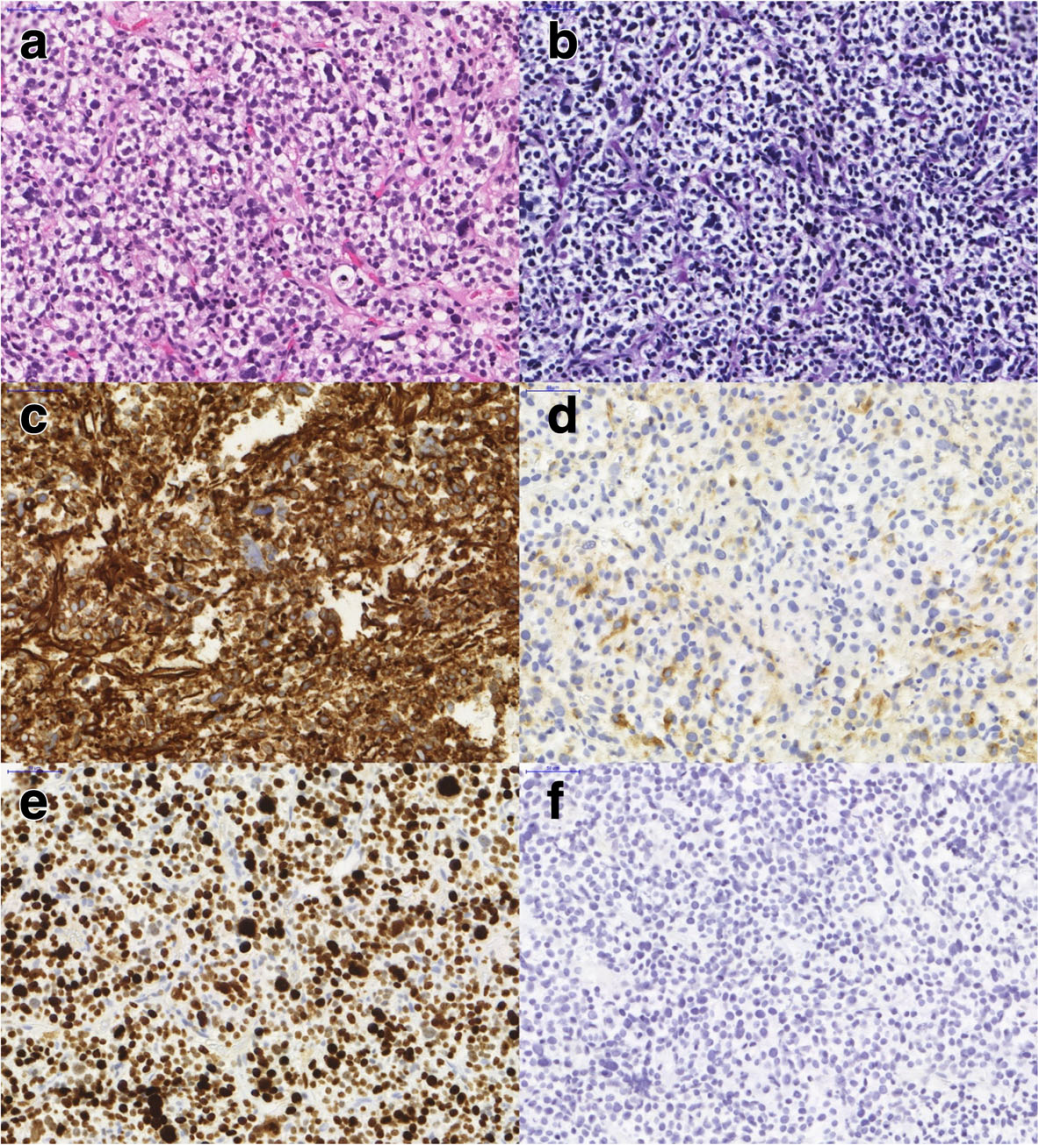
Histologic appearance of the PEComa: **a** hematoxylin-eosin stain revealing a pleomorphic tumor. The tumor cells did not produce PAS-positive material (**b**) and showed strong expression of vimentin (**c**) with at least partial co-positivity for MelanA (**d**). Proliferative activity in MIB1/KI-67 stain was very high (**e**) and cytokeratin reaction was negative (**f**). 40× magnification

In July 2015 the histopathological workup of a repeated biopsy showed a pleomorphic epitheloid tumor with small to medium sized cells. On immunohistochemistry, the tumor cells expressed vimentin and melan-A, but were negative for cytokeratin. Proliferative activity was very high measured by MIB-1/KI-67 (Fig. 2). A diagnosis of PEComa of the liver was clearly established. The lesion was classified as a malignant PEComa according to the Folpe classification because of its size (> 5 cm) and more than 1 risk factor (high proliferative activity, nuclear pleomorphism, multinuclear giant cells).

**Fig. 3:**
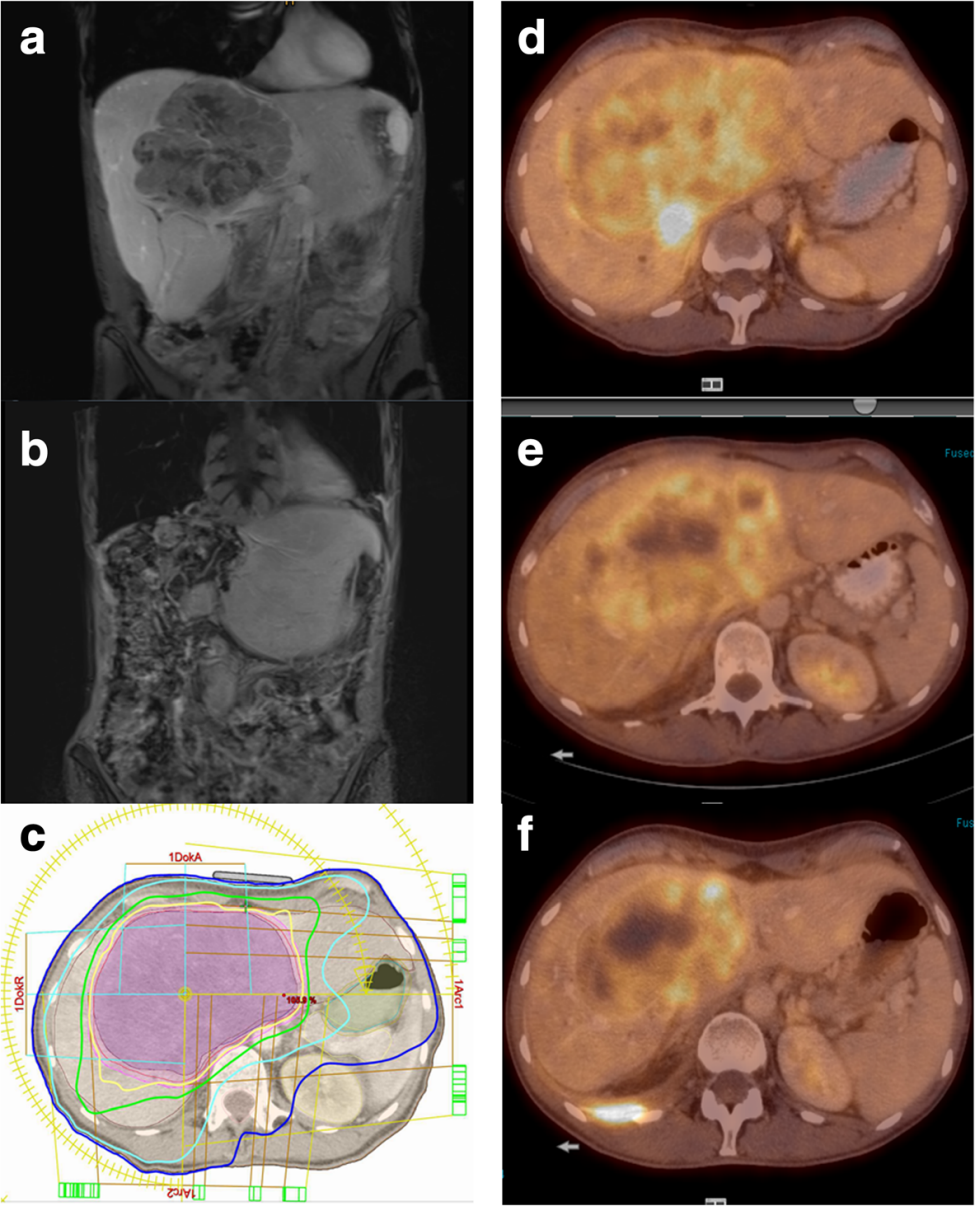
MRI and PET/CT images: **a** MRI images showing a T1 hypointense, heterogeneous mass (1280 ccm^3^) with peripheral contrast enhancement and washout in segments 1 and 4 to 8 before SBRT, **b**) MRI images after extended right hepatetctomy, **c**) Radiotherapy treatment plan (isodose lines: 57 Gy, 48 Gy, 30 Gy, 12 Gy in yellow, green, light blue, and dark blue respectively), **d**) PET/C before stereotactic body radiotherapy (SBRT), **e**) PET/CT during SBRT **f**) PET/CT after SBRT

Magnetic resonance imaging (MRI) of the abdomen showed progression of the tumor to 1280 cm^3^ within 26 days after initial CT (Fig. 3). FDG-PET/CT showed no evidence of distant metastasis. Due to tumor size and invasion of the inferior vena cava, the tumor was deemed unresectable and the multidisciplinary tumor board recommended neoadjuvant radiotherapy. To achieve high, ablative doses a stereotactic body radiotherapy (SBRT) technique was chosen consisting of 8 fractions of 7.5Gy prescribed to the 80% isodose to a total dose of 60Gy (biologically effective dose 105Gy; BED = total dose*(1+ dose per fraction/α/β) for α/β = 10 Gy) given every other day. The patient was immobilized in a vacuum bag using abdominal compression. Radiotherapy planning was based on a 4D planning CT and MRI resulting in a target volume (PTV) of 1944 cm^3^. A treatment margin of 4 mm was used around the GTV to create the PTV. Dose constraints for organs at risk were used as published elsewhere [17]. The whole liver had a volume of 3709 cm^3^ and 3330 cm^3^received a dose below 15 Gy. Treatment toxicity was limited to a slight elevation of transaminases (grade 1 and 3).

To evaluate efficacy of treatment, FDG-PET/CT was repeated (week 8, 17) showing no change in tumor size, but a reduction of SUV_max_ at 8 weeks and substantial reduction in tumor volume (49%) at 17 weeks post SBRT. At the multidisciplinary tumor board, the patient was deemed operable because of the significant downsizing effect. A complete resection was performed 21 weeks after radiotherapy consisting of an extended hemihepatectomy [segments 1, 4-8), tangential resection of the retrohepatic cava, and biliodigestive anastomosis via end to side technique (Roux-Y). There were no intraoperative complications. The postoperative course was complicated by sepsis, wound healing problems and weight loss. Histological and immunochemistry findings were consistent with the diagnosis of PEComa showing more than 80% necrosis corresponding to a pathological response after SBRT. Surgical margins were negative.

At last follow-up 21 months after therapy, MRI showed neither local nor distant tumor recurrence. The patient was in stable condition (ECOG 1) with no symptoms of late radiation toxicity.

## Discussion

The term “PEComa” unifies a multitude of rare, histologically diverse tumor types, which can occur in almost every organ with a predominance of pelvic organs.

In the presented case a PEComa of the liver was diagnosed by CT scan and subsequent biopsy. Following the criteria outlined by Folpe et al. the lesion was classified as a malignant hepatic PEComa demonstrating a rapid progression almost doubling its size within one month. Additional imaging with MRI revealed an inhomogeneous, intrahepatic mass with marked restriction of diffusion, compression of the biliary tract and intrahepatic cholestasis. A PET/CT that was ordered for staging purposes and for radiotherapy planning showed a huge liver lesion with moderate to marked, inhomogeneous PET tracer enhancement. After pathologic workup resulted in the diagnosis of PEComa the patient was treated with neoadjuvant SBRT followed by resection.

Few, small case series describe morphologic imaging characteristics of hepatic PEComa on CT, MRI or ultrasound. Radiologic characteristics that can differentiate between a benign liver lesion (e.g. angiomyolipoma) and PEComa with malignant potential have not been identified so far. In summary hepatic PEComa frequently present as a solitary, well-circumscribed heterogeneous mass in patients with no underlying liver disease. Contrast-enhanced CT and MRI showed the lesions were significantly and heterogeneously enhanced on arterial phase, less enhanced on portal venous phase, and slightly hypodense on delayed phase [18–20]. These features can make it difficult to distinguish PEComa and hepatocellular carcinoma by enhancement pattern and final diagnosis always needs to be obtained by histology.

The few published reports on the role of FDG-PET/CT are discordant regarding its role in diagnosis and staging. It is supposed that benign PEComas are not showing a significant tracer uptake. A report of 12 patients by Young et al. showed that benign LAM lesions were not detectable by FDG PET/CT [21]. Other reports demonstrated FDG-PET avidity in hepatic, adrenal und retroperitoneal AML [22–25]. Unfortunately, since the tumors were not classified according to the FOLPE-criteria, it is unclear which subtypes of PEComas were FDG-avid. Several studies report that both primary and metastatic malignant PEComas (NOS) of the uterus, retroperitoneum and bone were detectable by FDG-PET/CT [13–18, 26–28]. Sun et al. described FDG-uptake of metastases of a malignant uterine PEComa in multiple sites including lung and liver with maximum standardized uptake values (SUV max) between 6.5 and 12.1.

Precise therapeutic guidelines do not exist. Resection with clear margins is the treatment option of choice for resectable tumors, because even benign lesions can show aggressive growth tendency. Additional or further treatment options such as chemotherapy, therapy with mTOR-Inhibitors or radiation therapy have to be explored more intensively.

Hepatic manifestation of PEComa is rare. Table 1 shows 21 cases reported in the literature. All except one case were resectable and the primary treatment was operation. Of 18 cases with available follow up information, 14 were recurrence free, although follow up was short and one patient died shortly after the operation. Another study reported on 94 cases which were treated by surgery [29]. No recurrence was observed during the follow up period. The authors recommend surgery to avoid spontaneous rupture and compression if the tumor is larger than 5 cm.

**Table 1:** Reported cases of hepatic PEComa. Review of the literature [34–49]

No	Author	Age/sex	Tx	Follow-up [months]	Outcome	Imaging of primary tumor	Imaging for follow-up
1	Sheng et al.	55/M	OP	12	NED	MRI	MRI
2	Paiva et al.	51/F	OP	25	NED	CT	n/a
3	Sanchez-Perez et al.	32/F	OP	n/a	n/a	CT/MRI	n/a
4	Cheung et al.	53/F	OP	12	NR	CT	CT
5	Zhao et al.	58/M	OP	9	NR	US, CT	n/a
6	Patra et al.	50/F	OP	24	NED	CT	n/a
7	Fang et al.	56/F	OP	24	NED	CT	n/a
8	Ameurtesse et al.	63/F	OP (not R0)	9	NED	US, CT, MRI	n/a
9	Khaja et al.	51/F	n/a	n/a	n/a	US, FDG-PET/CT, MRI	n/a
10	Jafari et al.	53/F	OP	14	NED	CT, MRI	CT, MRI
11	Zhang et al.	63/F	OP	8	NED	CT	n/a
12	Zimmermann et al.	53/M	OP	17	NED	US, FDG-PET/CT, MRI	n/a
13	Panahova et al.	38/M	OP	6	NR	US, CT, MRI	
14	Ahn et al.	36/F	OP	3	NED	CT	n/a
15	Della Vigna et al.	46/F	OP	n/a	n/a	US, MRI	n/a
16	Maebayashi et al.	58/M	OP	60	NED	CT, MRI	n/a
17	Liu et al.	25/F	OP	12	NR	US, CT, MRI	n/a
18	Yu et al.	41/F	OP	9	NED	US, CT	n/a
19	Jurado et al.	45/F	OP	n/a	n/a	CT	n/a
20	Selvaggi et al.	42/M	OP	< 1	fatal	CT	n/a
21	Akitake et al.	36/F	OP	18	NED	US, CT, MRI	n/a

*Tx* therapy, *NED* no evidence of disease, *US* ultrasound

There is limited data for the use of radiotherapy in the treatment of PEComa. Only four cases are reported in the literature and no data on the radiosensibility of PEC is available. In one case of a PEComa of the upper extremity, RT of 50 Gy was given preoperatively [30]. The other cases are in the postoperative setting using doses of 45-60 Gy. One patient experienced recurrent disease after 6 months, the others had no evidence of disease (NED) after 17 and 15 months [31–33].

We present the first case of neoadjuvant SBRT with an ablative dose in a malignant liver PEComa and achieved conversion to resectability with this strategy. Although surgeons were concerned about possible intraoperative complications caused by radiotherapy effects on the liver parenchyma, no intraoperative complications, such as extensive bleeding, occurred. PET/CT was used for staging purposes and for evaluation of treatment response of the FDG avid tumor. This seems to be a good strategy for PEComas showing PET tracer positivity at initial imaging.

## Conclusion

PEComa is a rare tumor that can display both benign as well as malignant characteristics. Resection is the treatment option of choice for resectable cases. We safely applied SBRT as a neoadjuvant therapeutic strategy to turn an inoperable primary hepatic PEComa into a resectable lesion. Preoperative radiotherapy is a potential treatment paradigm for inoperable PEComas of the liver as well as in other anatomic regions.

## Abbreviations

AML: angiomyolipoma
CCST: clear cell sugar tumor
LAM: lymphangioma
NOS: not otherwise specified PEComa
PEComa: Perivascular epithelioid cell tumor
PET/CT: positron emission tomography
PTV: planning target volume
SBRT: stereotactic body radiotherapy
